# Modeling the Phase Transition in Hydrophobic Weak Polyelectrolyte Gels under Compression

**DOI:** 10.3390/gels9030259

**Published:** 2023-03-22

**Authors:** Alexander D. Kazakov, Varvara M. Prokacheva, Oleg V. Rud, Lucie Nová, Filip Uhlík

**Affiliations:** Department of Physical and Macromolecular Chemistry, Faculty of Science, Charles University, 12800 Prague, Czech Republic

**Keywords:** polyelectrolyte hydrogels, simulations, desalination, hydrophobic gels, weak polyelectrolytes, volume-phase transition

## Abstract

One of the emerging water desalination techniques relies on the compression of a polyelectrolyte gel. The pressures needed reach tens of bars, which are too high for many applications, damage the gel and prevent its reuse. Here, we study the process by means of coarse-grained simulations of hydrophobic weak polyelectrolyte gels and show that the necessary pressures can be lowered to only a few bars. We show that the dependence of applied pressure on the gel density contains a plateau indicating a phase separation. The phase separation was also confirmed by an analytical mean-field theory. The results of our study show that changes in the pH or salinity can induce the phase transition in the gel. We also found that ionization of the gel enhances its ion capacity, whereas increasing the gel hydrophobicity lowers the pressure required for gel compression. Therefore, combining both strategies enables the optimization of polyelectrolyte gel compression for water desalination purposes.

## 1. Introduction

Fresh water production is one of the most pressing issues for modern humanity [1]. Seawater desalination offers an option to satisfy the increasing demand of fresh water. There are many technologies that can be divided into two groups, namely thermal-based (e.g., multistage flash distillation) and membrane-based (e.g., reverse or forward osmosis) methods. Each group of methods has advantages and disadvantages. For example, the traditional thermal-based desalination is simpler and cheaper as itself, but associated with high energy costs, whereas the membrane-based technology has lower operating costs, but requires very expensive membranes [2,3].

An alternative desalination method using polyelectrolyte hydrogels has been proposed recently [4,5,6]. It can be viewed as a modification of the forward osmosis (FO) method, where the gel acts both as the draw solute and as the separation membrane [5,7].

A hydrogel is a gel in which the swelling agent is water [8]. Here, we use this term for a network of cross-linked polymer chains, which swells in aqueous solutions. The swelling equilibrium is determined by the interplay between the network elasticity and the osmotic pressure created by the solvent penetrating the network. Hydrogels are known as super-absorbers for their ability to absorb huge amounts of water and can increase their initial (dry) volume by three orders of magnitude [9]. Thanks to these properties, hydrogels have a wide range of applications, including personal care (disposable diapers) [10], agriculture (improving soil water retention) [11] and bioengineering (self-healing materials) [12,13], in addition to water desalination [14,15]. Hydrogels with controllable features are also known as “stimuli-responsive” materials [16,17] because they can change their properties in response to different external stimuli, such as pH, salinity, or electric fields.

The stimuli-responsive properties of *polyelectrolytes* (PEs) result from the interplay between long-ranged electrostatic forces, short-ranged steric interactions and the entropic elasticity of polymer chains. In a solution, polyelectrolytes show not only large continuous volume changes during swelling–deswelling, but also first-order volume-phase transitions (VPT). These phenomena were theoretically first predicted and explained for weakly charged strong polyelectrolytes by Borue and Erukhimovich [18] and later extended to solutions of linear PE chains [19,20,21], PE stars [22,23,24] and PE combs [25]. Nevertheless, experimental evidence of phase transitions and microphase separation in PEs has also been reported in several studies [26,27,28].

VPT in gels was already theoretically predicted by Dušek [29] using Flory theory in 1968 and later experimentally demonstrated [30]. VPT and microphase separation in gels upon changes in temperature and solvent composition were then studied many times, e.g., [31,32,33,34,35].

Upon varying the number of fixed ionic groups and the Coulomb coupling parameter, Mann et al. demonstrated the co-existence of microphases in polyelectrolyte hydrogels, namely a sausage-like state, a state with nodes (condensation nucleus), and a pearl-necklace state [36]. Furthermore, VPT can also be induced by increasing the temperature (and subsequently changing the solvent quality) in pNIPAM-gels incorporated with acrylic acid groups [37,38], by changing the pK of phenylboronic acid-based hydrogels [39,40,41], by inducing mechanical uniaxial stress in pNIPAM-gels [42], and even by adding salt [43,44,45,46,47].

Recently, by applying the mean-field analytical theory [48], we demonstrated that the phase transition allows for a higher compression rate for hydrophobic polymer hydrogel compared to hydrophilic gel. Thus, theoretically, a larger volume of a solution may be extracted from a hydrophobic polymer gel during VPT without requiring high pressures. Although some authors have reported the experimental feasibility of salt water desalination using a poly(acrylic acid) hydrogel [4], which can be reused for several cycles even when compressed to 80 bars [5], others have argued that the hydrogel reversibility is limited to pressures of a few atmospheres [49,50]. In this context, we hypothesize that operating hydrophobic polymer gels at VPT and optimizing other conditions for lower pressure may avoid gel damage, thereby enabling the reuse of these gels in a large-scale water desalination process.

In this study, we aimed to investigate the structure of weak polyelectrolyte hydrogels upon their compression at different conditions: pH−pK difference, solvent quality (or gel hydrophobicity), and salinity. For this purpose, we performed coarse-grained simulations and their comparison with an analytical mean-field theory [48]. Our findings complement the understanding of VPT in weak polyelectrolyte hydrophobic gels and provide insight into the internal structure of such gels at the phase transition region, with potential implications for the water desalination process by means of lowering the working pressure and subsequently mitigating the problem of damage of the hydrogel network.

## 2. Results and Discussion

### 2.1. Gel Model

We model the gel as a diamond-like network of polymer chains. Each chain comprises N=50 monomer units. The network is in equilibrium with a reservoir of aqueous (bulk) solution at specific concentration $c_{s}$ of positive and negative monovalent ions, denoted as ${\mathrm{Na}}^{+}$ and ${\mathrm{Cl}}^{-}$.

Each monomer unit (bead) of the network carries an acidic pendant, which can be either charged or uncharged, according to the following reaction:

(1) $$\mathrm{HA}\overset{K}{⇄}A^{-}+H^{+},$$

where *K* is the ionization equilibrium constant.

Due to this reaction, the gel is partially charged, and the ionization degree α varies depending on the parameters of the environment

(2) $$\alpha =\frac{\rho_{A^{-}}}{\rho_{A^{-}}+\rho_{\mathrm{HA}}}$$

where $\rho_{A^{-}}$ and $\rho_{\mathrm{HA}}$ are the densities of charged and non-charged monomer units.

At the same time, each monomer unit carries a hydrophobic pendant, characterized by a hydrophobicity parameter. Depending on the simulation approach, as a hydrophobicity parameter we use either ε (the Lennard–Jones interaction parameter) or χ—the Flory–Huggins parameter.

The two approaches used in our study are the: coarse-grained (CG) and mean-field (MF) simulation modeling.

#### 2.1.1. Coarse-Grained (CG) Model

In this model, we consider a network of 16 polymer chains, which are interconnected via periodic boundary conditions to emulate the bulk hydrogel. We account for three simultaneous processes: (1) the mechanical movement of all the particles, (2) the ionization reaction of the gel monomer units and (3) the grand-canonical exchange of ${\mathrm{Na}}^{+}$ and ${\mathrm{Cl}}^{-}$ ions with a reservoir. We used Langevin molecular dynamics to sample process (1) and and Monte Carlo for sampling the grand reaction ensemble [51,52] accounting for processes (2) and (3).

The volume of the simulation box $V_{\mathrm{gel}}$, the hydrophobicity parameter ε, the chemical potentials of the ions and the ionization constant *K* are the input parameters for the simulation. The output are the averages of pressure, number of ions $n_{{\mathrm{Na}}^{+}}$ and $n_{{\mathrm{Cl}}^{-}}$ in the simulation box, ionization degree, α from Equation (2) and other quantities of interest. We also determine the pressure of the bulk solution by running a separate simulation of the same system, but without the gel.

The difference of the obtained pressure and the pressure of the bulk solution gives the pressure difference *p*, that must be applied to the hydrogel using a solvent permeable piston to achieve the desired gel (number) density ρ, that we define as $\rho =\left(16N+8\right)/\left(N_{A}V\right)$, where *N* is the number of monomers in a single gel strand (N=50 in our simulations), *V* is volume of the simulation box and $N_{A}$ is the Avogadro constant. We use density ρ as the independent variable, which we set by varying the simulation box volume.

#### 2.1.2. Mean Field (MF) Model

The mean-field approximation is based on the classical lattice Flory theory of polymers. In this approximation, a gel network strand of the length *N* was considered interacting with a mean-field produced by the other components of the system: water, salt ions, and the rest of the gel [22,53]. The free energy of a chain, *F*, consists of three independent terms: The conformational entropy of a uniformly extended chain $F_{\mathrm{conf}}$, short-range non-electrostatic interactions (which account for the hydrophobicity of the gel network) $F_{\mathrm{int}}$, and the ionic contribution $F_{\mathrm{ion}}$

(3) $$F=F_{\mathrm{conf}}+F_{\mathrm{int}}+F_{\mathrm{ion}}$$

The entire formula for the free energy is a function of the gel density ρ and contains $c_{s}$, χ and pH−pK as parameters. We obtain the pressure applied to the gel as a derivative of the hydrogel free energy with respect to the gel molar volume

(4) $$p\left(\rho \right)=-\frac{\partial F(\rho )}{\partial (1/\rho )}.$$

The expanded view of the expression (3) can be found in Section 4.3.

#### 2.1.3. Maxwell Construction

**Figure 1:**
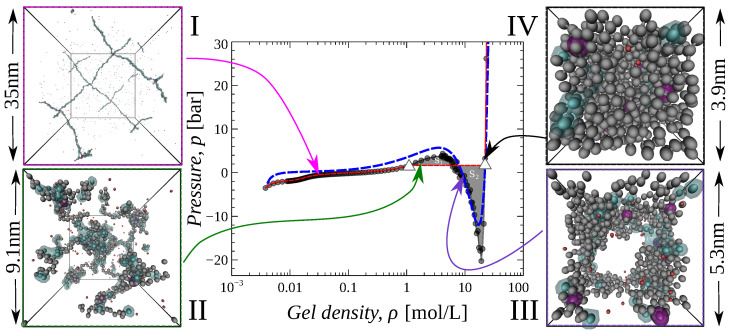
Pressure-extension curves of a hydrophobic gel with pH−pK=1, ε=0.5$k_{B}T$, reservoir salt concentration $c_{s}=0.01$ mol/L with inserted simulation snapshots at the following gel densities: (I) ρ=0.03, (II) 1.78, (III) 8.99, and (IV) 22.06 mol/L. The black curve results from simulations and the red one from the Maxwell construction over the black curve. The blue dashed curve corresponds to a fit of simulation data by analytical theory equation (*vide infra*). White triangles represent bimodal points. The blue clouds indicate charged segments of the gel. The magenta spheres show the nodes of the gel. The red and blue spheres represent counter- and co-ions, respectively. For more snapshots, see the ESI (Figures S4 and S5).

Under some conditions, the solution of Equation (4) and the results of simulations lead to an unphysical outcome, that is, a decrease in the applied pressure with the gel compression, as shown by the loop between the white triangles of the black pressure-extension curve in Figure 1. This resembles the behavior of models of real gases, where competition between attractive and repulsive interactions results in phase separation. This similarity allows us to draw an analogy between the hydrogel phase transition theory and the van der Waals theory of the liquid–vapor phase transition [54,55] when the analytical description of liquid/vapor (in our case hydrogel) behavior is represented by unrealistic, non-monotonic functions of applied pressure on density.

To plot a realistic (monotonic) pressure-extension dependence we use the so-called Maxwell construction [56,57]. The Maxwell construction allows us to replace the van der Waals loops by horizontal lines, which are drawn such that the areas, $S_{1}$ and $S_{2}$, bounded by the line and the loop from above and below are equal. The Maxwell construction is shown in Figure 1 by the red dashed line and it shows a monotonous increase of the pressure versus compression. This dependence consists of three parts: first, at low densities, *p* increases with density, then it reaches a plateau and remains constant, then at a certain high density it starts to increase abruptly.

The presence of the plateau in the p(ρ) dependence indicates the first order phase transition happening in the gel during compression.

### 2.2. Pressure Extension Curve

**Figure 2:**
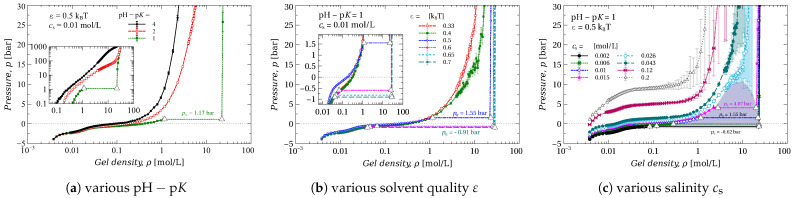
The pressure *p* applied to the gel as a function of gel density ρ at different values of (**a**) pH−pK difference, (**b**) solvent quality ε and (**c**) salt concentration $c_{s}$. White triangles mark the borders of the two-phase region. The shaded area highlights the two-phase area limited by binodals. The original data (with loops) and Maxwell construction details are provided in the ESI (Figures S1 and S2).

We studied gel compression under different conditions by varying the solvent quality (ε parameter), salt concentration $c_{s}$, and pH−pK difference. Figure 1 and Figure 2 show the corresponding dependencies of the pressure difference *p* on the gel density ρ.

The dependencies presented in the figures can be divided into two types, depending on whether: (i) the pressure increases monotonically with gel density or (ii) the p(ρ) contains a plateau due to the Maxwell construction. In the latter case, the dependencies consist of three regions: (a) in the low-density region, the compression reacts as a smooth increase of pressure *p*. This region corresponds to a single phase of swollen gel (e.g., inset I in Figure 1); (b) in the high-density region, compression causes a sharp increase of *p*, which corresponds to the compression of a single phase of collapsed (dry) gel (e.g., inset IV in Figure 1); (c) between these two regions, the pressure remains constant. We assume that this region corresponds to the coexistence of two phases of the gel (e.g., insets II and III in Figure 1) and we refer to the value of the pressure in this region as transition pressure $p=p_{\mathrm{tr}}$.

Although the pressure is constant in the two-phase region, the average gel density, ρ, varies with the compression. The average is calculated over the two coexisting phases, so the fractions of both phases define the value of ρ according to the lever rule [57].

In the two-phase region, the hydrogel has a domain structure, as shown in insets II and III in Figure 1. In inset II, the domains are small and interconnected through a large number of stretched chains, but in the more compressed state (inset III), the size of the domains is bigger, whereas the fraction of stretched chains is smaller. The physics of such a phase transition lies in the interplay between electrostatic and hydrophobic interactions. Electrostatic interactions are long-ranged and repulsive, whereas hydrophobic ones are short-ranged and attractive. The compression forces the gel beads to discharge; the discharged beads stick together and form dense domains.

The pH−pK difference plays a key role in the formation of the gel structure. In Figure 2a, it is seen that the gel with pH−pK=4 and 2 does not exhibit phase transition, whereas the gel with pH−pK=1, by contrast, passes through the plateau and thus exhibits phase transition (VPT).

The change of ε from 0.33 to 0.7$k_{B}T$ models solvent quality deterioration from Θ to poor solvent, respectively [58]. The increase of ε causes VPT, and moreover leads to a decrease of transition pressure $p_{tr}$, and to a broadening of the transition region, see Figure 2b.

The change of the surrounding salinity $c_{s}$ affects the phase transition as well, see Figure 2c. In order to show it, we varied the salinity from $c_{s}=0.2$ mol/L to low $c_{s}=0.002$ mol/L. At high salinity, the pressure gradually increases with gel compression. At $c_{s}=0.026$ mol/L, the corresponding p(ρ) curve seems to be passing through a critical point. With a further decrease of the salt concentration, VPT occurs, at transition pressures of a few bars. The transition pressure decreases with the decrease of $c_{s}$.

### 2.3. Phase Diagram

The two-phase region can be defined by drawing a line (binodal) through the white triangles in pressure-extension curves at different salt concentrations in Figure 2c. The gel has two phases inside the binodal and only one outside, which is either swollen (at low ρ) or collapsed (at high ρ), as shown in Figure 2c where the shadowed area represents the two-phase region.

**Figure 3:**
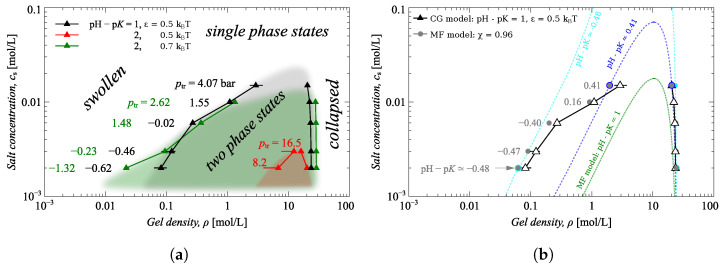
Phase diagrams of a hydrophobic gel in the coordinates salt concentration $c_{s}$ versus gel density ρ. (**a**) Comparison of simulation results at different values of solvent quality ε and pH−pK. The values of $p_{tr}$ are the results of Maxwell construction (see Figure S2 in ESI). (**b**) Comparison of simulation results at pH−pK=1 and ε=0.5 with analytical theory (MF) results at solvent quality χ=0.96 and different pH−pK. The gray dots are the points belonging to binodals calculated by the MF model for different pH−pK as indicated. Three of these binodals are plotted by dotted lines (green, blue and cyan).

The two-phase region is also highlighted in the phase diagram in Figure 3a, which is plotted in the coordinates: salinity $c_{s}$ versus gel density ρ. Figure 3a shows how the state of the gel depends on the salinity and the gel density. The states marked by black triangles in this figure are the same states as those marked by triangles in Figure 2c, i.e., these points belong to a binodal line separating the single phase and two-phase states of the gel. The shadowed areas are the guides to eyes marking the two-phase regions of the phase diagram.

Figure 3a also shows that the increase of the pH−pK narrows the two-phase region and increases the transition pressure $p_{\mathrm{tr}}$, whereas the increase of ε broadens this region and decreases the $p_{\mathrm{tr}}$. Both of these effects are manifested by the fact that the binodal corresponding to pH−pK=1 and $\varepsilon =0.5k_{B}T$, and the binodal for pH−pK=2 and $\varepsilon =0.7k_{B}T$ are almost lying on top of each other.

Figure 3b compares simulations results for pH−pK=1 and $\varepsilon =0.5k_{B}T$ with the results of analytical theory. To enable this simple, one-to-one comparison, between simulations (black curve) and analytical theory solutions (green curve), we applied the settings used in the simulations to the analytical theory, namely pH−pK=1 and solvent quality χ=0.96 (that approximately corresponds to $\varepsilon =0.5k_{B}T$). In order to obtain the correspondence, we fitted the coarse-grained model simulation data by the mean-field formula Equation (4). For details of the fitting procedure, see the ESI Section S3.

The MF model predicts the two-phase region to be much narrower than that provided by the CG model (compare the green dashed line with the black line with triangles). Nevertheless, the higher the salt concentration, the better both models agree, and both binodals (black and green lines) seem to be passing through the same critical point. This is due to the more screened electrostatic interactions at high salinity. The agreement between the CG and MF models at low salinity can be improved by allowing pH–p*K* to be a fitting parameter (“effective” pH–p*K*). This correction of the MF model accounts for its oversimplified description of electrostatics.

The gray dots on the plot are the results of fitting the CG model data by the MF theory by varying pH–p*K* parameters (and fixed χ=0.96). Each pair of gray points, composing the binodal, is calculated by the MF model for different (fitted) pH–p*K*, as indicated in the plot (numbers printed in gray). It is evident that the MF model binodal line follows the binodal of the CG model providing that the “effective” pH–p*K* decreases with decreasing salinity. This decrease of effective pH–p*K* can be explained by the screening of electrostatic interactions. As stated above, the MF model underestimates the ionic contribution, accounting only for the Donnan partitioning effect on gel ionization, whereas the CG model accounts for the direct electrostatic interactions and, therefore, for the screening by the mobile ions. The lower the salinity, the stronger the direct electrostatic interactions; therefore, the higher energy needed for ionization of the neighboring beads of the gel network, which manifests itself as a dependence of the effective p*K* on the salt concentration.

Based on Figure 2 and Figure 3, we conclude that VPT lowers the pressure needed for the compression of the gel. As a result, a hydrophobic gel becomes more suitable for water desalination than a hydrophilic one. However, the ion capacity of the gel, that is, the number of salt ions that the gel can absorb, is determined by its ionization degree.

### 2.4. Ionization Degree of the Gel

**Figure 4:**
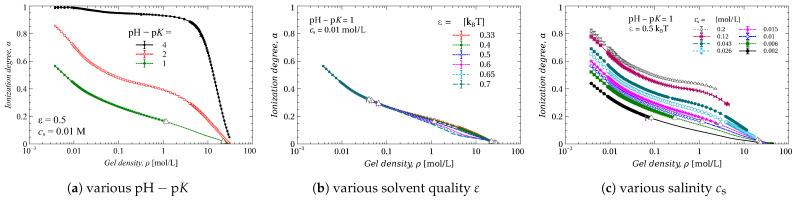
Ionization degree α of the gel and as a function of gel density ρ for different (**a**) pH−pK, (**b**) solvent quality ε and (**c**) salt concentration $c_{s}$. White triangles define the borders of the two-phase area.

Figure 4 shows the average gel ionization degree α as a function of gel density ρ. This dependence is displayed in the same manner as in Figure 2: for various pH−pK (Figure 4a), various ε (Figure 4b), and various $c_{s}$ (Figure 4c). Although different phases should have different ionization degrees [23,25], the phase separation does not show up in the dependencies of the average gel ionization; all the dependencies look smooth. We highlighted the points belonging to binodals as in Figure 2, by white triangles.

All three plots show that the compression discharges the gel. The increase of pH−pK significantly increases the gel ionization; this is quite an obvious pattern, which shows up in the growth of pressure needed for the gel compression (compare Figure 2 and Figure 4). By contrast, varying the solvent quality, ε, affects the ionization degree only weakly, which becomes noticeable at a gel density ranging from 0.1 to 10 mol/L (Figure 4b). The salinity of the bath, $c_{s}$, affects the ionization of the gel significantly (Figure 4c). The effect of salinity manifests itself in the decrease of the “effective” p*K* [57]. The presence of salt ions screens the electrostatic interactions of the neighboring chain beads, which, in turn, lowers the energy of their ionization. In other words: the higher the salinity, the higher the ionization.

### 2.5. Ion Transfer

**Figure 5:**
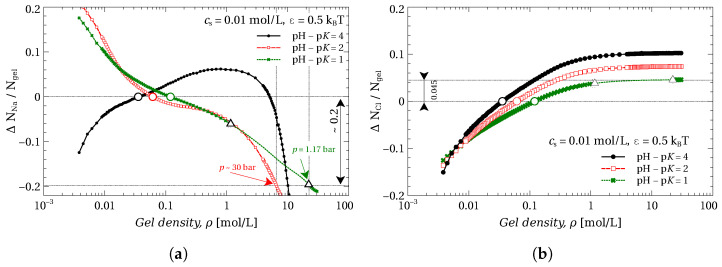
Change in the number of ${\mathrm{Na}}^{+}$ (**a**) and ${\mathrm{Cl}}^{-}$ (**b**) ions in the volume $V_{0}$ normalized by the number of gel segments $N_{\mathrm{gel}}$ as a function of gel density ρ for different pH−pK. White triangles define the borders of the two-phase area. Circles represent free swelling equilibrium states, where the applied pressure is zero.

The presence of the charges on the gel chain implies the absorption of counterions into the gel and the rejection of co-ions. Figure 5a demonstrates how this phenomenon affects the transfer of ${\mathrm{Na}}^{+}$ ions during the gel compression. The plotted value is the change of the number of ${\mathrm{Na}}^{+}$ ions in a volume $V^{0}$ the gel is compressed in (calculated per bead of the gel). $V^{0}$ is the gel volume at free swelling equilibrium, i.e., at zero applied pressure. Thus, the change in the number of ${\mathrm{Na}}^{+}$ ions in this volume is

$$\Delta N_{{\mathrm{Na}}^{+}}=N_{{\mathrm{Na}}^{+}}^{\mathrm{gel}}+c_{s}\left(V^{0}-V^{\mathrm{gel}}\right)-N_{{\mathrm{Na}}^{+}}^{0},$$

where the first term, $N_{{\mathrm{Na}}^{+}}^{\mathrm{gel}}$ is the number of ${\mathrm{Na}}^{+}$ ions in the gel, the second term is the number of ions in the outer volume, which is left after compression ($V^{\mathrm{gel}}$ is the gel volume at a specific applied pressure), and $N_{{\mathrm{Na}}^{+}}^{0}$ is the number of ${\mathrm{Na}}^{+}$ in the gel at free swelling equilibrium (before compression). The positive value of $\Delta N_{{\mathrm{Na}}^{+}}$ implies that the ions are transferred from the bath to the volume $V^{0}$, whereas the negative value of $\Delta N_{{\mathrm{Na}}^{+}}$ means that the ions are pushed out to the bath.

Figure 5a shows that, in general, the value $\Delta N_{{\mathrm{Na}}^{+}}$ is a non-monotonic function of compression. For the gel with high pH−pK=4 at low gel densities, which corresponds to almost constant ionization of the gel (compare the black lines in Figure 4 and Figure 5), the compression of the gel leads to the accumulation of ${\mathrm{Na}}^{+}$ ions in the volume $V^{0}$. At higher compressions, the ${\mathrm{Na}}^{+}$ ions are released to the bath. This effect can be explained [59] as follows: While the gel does not discharge, the counterions remain inside the gel, thus their density in gel increases with compression. However, when the discharge rate of the gel becomes sufficiently high, the counterions are released from the gel, and thus from the volume $V^{0}$ as well.

The larger the change in $\Delta N_{{\mathrm{Na}}^{+}}/N_{\mathrm{gel}}$ upon compression in the two-phase region, the more ions are transferred and thus the more useful such a system is for desalination purposes. Figure 5a shows that employing the VPT in gel allows to reach rather high values of $\Delta N_{{\mathrm{Na}}^{+}}/N_{\mathrm{gel}}$, using at the same time rather small pressures. For instance, the compression of the gel with pH−pK=1 allows us to transfer $\Delta N_{{\mathrm{Na}}^{+}}/N_{\mathrm{gel}}≃0.2$ mol of ${\mathrm{Na}}^{+}$ ions per mol of gel beads using the pressure not exceeding $p=p_{tr}=1.2$ bar. The same result for the gel with pH−pK=2 already requires a pressure of about 30 bar (compare with Figure 2) and it is even higher for the gel with pH−pK=2.

Because the concentration of co-ions ${\mathrm{Cl}}^{-}$ in the gel is always lower than in the bath, their number in the volume $V^{0}$ always increases with gel compression, disregarding whether the gel is discharging or not. These dependencies are present in Figure 5b and are quite different from those of the Figure 5a. This is apparently due to gel discharging (being protonated) during compression, which implies that the gel consumes H^+^ ions, which are not considered explicitly in our model. If we plotted the change of the total number of the counter-ions, i.e., Na^+^ minus the consumed H^+^ ions, we would observe the same dependencies as for the co-ions, ${\mathrm{Cl}}^{-}$ ions.

The VPT described in this work is associated with the gel discharge; therefore, the compression of the gel always produces some water ions, ${\mathrm{OH}}^{-}$ or $H^{+}$. The compression of a polyacid gel will produce OH^-^ ions and release Na^+^ ions, and the compression of polybase gel will produce H^+^ ions and release ${\mathrm{Cl}}^{-}$ ions. The simultaneous compression of both types of gel, producing equal amounts of ${\mathrm{OH}}^{-}$ and $H^{+}$, would allow us to transfer both Na^+^ and Cl^-^ ions simultaneously and thus may be utilized for water desalination [60].

In Figure 5, we considered only a particular case of ion transport at rather low salinity, $c_{s}=0.01$ mol/L. Nevertheless, the VPT can by induced at the conditions of higher salinity by tuning the properties of the gel: i.e., the hydrophobicity, ε, and pH−pK.

## 3. Conclusions

We studied the VPT of a hydrophobic hydrogel, from swollen to collapsed states through intermediate states, in which two phases of the gel (swollen and collapsed) coexist in a proportion that varies with compression. At this intermediate state, the hydrogel has a domain structure. Some parts of the gel are collapsed and interconnected by stretched gel chains. In the two-phase region, compression of the gel occurs at a constant pressure and the domains sprawl. The two-phase region can be broadened by decreasing pH−pK (making gel less charged) and increasing ε (making gel more hydrophobic). Being more hydrophobic and less charged makes the gel less swollen, and finally collapsed; and oppositely, being less hydrophobic and more charged makes the gel more swollen. This could be used in facilitating the feasibility of water desalination by lowering the necessary pressure over hydrophilic gels and reducing the wear and fouling of gel and making the desalination device simpler.

Analytical theory underestimates the range of the two-phase region and overestimates the transition pressure $p_{\mathrm{tr}}$ because it does not account for direct electrostatic interactions to the overall free energy of the gel in the simple mean-field model. However, varying pH−pK with the salt concentration $c_{s}$ significantly improves the agreement between simulations and analytical theory results. The transition pressure difference decreases to only a few bars, and the two-phase region of the analytical theory nearly overlaps that of the simulation predictions. Therefore, the agreement between simulation and analytical theory results can be improved by introducing the relationship between pH−pK and the salt concentration $c_{s}$, thereby more accurately accounting for the ionic contribution.

## 4. Materials and Methods

As we mentioned above, our CG simulation approach accounts for mechanical movements, ionization reactions and ion exchange simultaneously. In order to simulate all these processes concurrently, we alternated short runs of Langevin dynamics (LD) and short runs of grand reaction ensemble Monte Carlo (MC) samplings.

Thus, after the system initialization and equilibration, we

1. Run Langevin dynamics making 150 integrations, each by $1.72\cdot 10^{-12}$ s, collecting samples of pressure values;
2. Run the Monte Carlo simulating 4096 reaction steps of the gel ionization reactions and ion pair exchanges, collecting the numbers of ${\mathrm{Na}}^{+}$ and ${\mathrm{Cl}}^{-}$ ions and the number of ionized beads $N_{A^{-}}$;
3. Repeat until we collect a sufficient number of independent samples to obtain accurate estimates of relevant quantity values.

Since we study the thermodynamic equilibrium, the particular lengths of the LD and MD blocks do not affect the results, but may have a significant impact on the computational efficiency. For a more detailed explanation of the theory behind the simulation setup, we address the reader to previous studies [52,59] and to electronic supporting information (ESI).

### 4.1. Langevin Dynamics

All explicit particles in the model are represented as points interacting via spherically symmetric potentials. We consider water as an implicit solvent, i.e., a structureless continuum characterized by relative dielectric permittivity, $\epsilon_{r}=80$.

All particles interact with each other via non-bonding interactions described by Lennard–Jones potential [61]:

(5) $$\begin{array}{c} V_{\mathrm{LJ}}\left(r\right)=\left\{\begin{array}{cc} 4\varepsilon \left[{\left(\frac{\sigma }{r}\right)}^{12}-{\left(\frac{\sigma }{r}\right)}^{6}+c_{\mathrm{shift}}\right] & \mathrm{if}\;r<r_{\mathrm{cutoff}}\\ 0, & r\geq r_{\mathrm{cutoff}} \end{array}\right., \end{array}$$

where *r* is the distance between particles, σ is the characteristic size of particles (we have chosen σ to be equal 0.35nm), ε is the depth of the potential well, $c_{\mathrm{shift}}$ ensures that the potential is continuous at $r_{\mathrm{cutoff}}$, and $r_{\mathrm{cutoff}}$ is the cut-off distance beyond which the potential is zero allowing for faster summation over pairs of particles.

The Lennard–Jones potential was originally developed to model liquid neon and not to describe the effective interactions of polymer segments. Although the interactions in macromolecular systems are more complex [62], this potential is widely used for non-bonding interactions in polyelectrotes [63,64].

We set the interactions between ions, as well as the interactions between ions and gel segments, to be purely repulsive. For that, we set the potential parameters $\varepsilon =k_{B}T$ and $r_{\mathrm{cutoff}}=2^{1/6}\sigma$. The attraction between the hydrogel particles, i.e., the effect of hydrophobicity of the gel network, was introduced by the attractive part of the Lennard–Jones potential, in particular by setting $r_{\mathrm{cutoff}}=3\sigma$, and varying ε.

Chemical bonds between gel particles are described by the finite-extension non-linear elastic (FENE) potential [65]:

(6) $$V_{\mathrm{FENE}}\left(r\right)=-\frac{1}{2}\kappa \Delta r_{\max}^{2}\ln\left[1-{\left(\frac{r}{\Delta r_{\max}}\right)}^{2}\right],$$

where κ is the magnitude of the symmetric interaction between two particles and $\Delta r_{\max}$ is the maximal bond length. In our simulations, we use parameters $\kappa =10k_{B}T/\sigma^{2}$, $\Delta r_{\max}=2\sigma$ [66].

The long-range electrostatic interactions are modeled via the Coulomb potential:

(7) $$V_{\mathrm{EL}}\left(r\right)=k_{B}T\frac{l_{B}}{r},$$

where $l_{B}$ is the Bjerrum length. In our model, we set $l_{B}≃0.7\;\mathrm{nm}=2\sigma$, which corresponds to the Bjerrum length in water at 300K. The electrostatic energy of the whole system was calculated by a Particle-Particle-Particle-Mesh (P3M) method [36].

The Langevin thermostat was used to ensure that the system was in thermal equilibrium with the heat bath at a temperature of T=300K [66].

### 4.2. Monte Carlo

Short runs of Monte Carlo simulations accounted: (i) for the ionization of monomer units (Equation (1)), and (ii) for the exchange of ${\mathrm{Na}}^{+}$ and ${\mathrm{Cl}}^{-}$ ion pairs with the reservoir, which we consider as a formal reaction:

(8) $$⌀\overset{K_{s}}{⇆}{\mathrm{Na}}^{+}+{\mathrm{Cl}}^{-},\;K_{s}=e^{\left(\mu_{{\mathrm{Na}}^{+}}+\mu_{{\mathrm{Cl}}^{-}}\right)/k_{B}T},$$

where $\mu_{{\mathrm{Na}}^{+}}$ and $\mu_{{\mathrm{Cl}}^{-}}$ are the chemical potentials of ${\mathrm{Na}}^{+}$ and ${\mathrm{Cl}}^{-}$ ions. In our model, ${\mathrm{Na}}^{+}$ and ${\mathrm{Cl}}^{-}$ differ only in the sign of the charge. We set $\mu_{{\mathrm{Na}}^{+}}=\mu_{{\mathrm{Cl}}^{-}}=\mu_{i}$ because we refer them both to the standard state of a one molar NaCl solution. In order to obtain the relation between the chemical potential, $\mu_{i}$, and the concentration, $c_{i}$, of the respective component, we run a separate simulation of the the grand-canonical equilibrium between the reservoir and the system without gel. In this simulation, we set up a particular value of $\mu_{i}$ and obtain the corresponding salinity $c_{s}$.

Due to low concentration of H^+^ ions near neutral pH, *c*_H^+^_ = 10^−7^ mol/L, we did not include explicit H^+^ ions in our model. Instead, we assumed that the reaction Equation (1) occurs only together with an exchange of ${\mathrm{Na}}^{+}$ ion with the H^+^ ion.

(9) $$H^{+}\overset{K^{\prime }}{⇄}{\mathrm{Na}}^{+},K^{\prime }=e^{(\mu_{H^{+}}-\mu_{{\mathrm{Na}}^{+}})/k_{B}T}$$

Thus, the reaction which we effectively model in our simulation is $\mathrm{HA}⇄A^{-}+{\mathrm{Na}}^{+}$, and the effective reaction constant $K\cdot K^{\prime }$ also accounts for the exchange of $H^{+}$ ion by ${\mathrm{Na}}^{+}$. The chemical potential of $H^{+}$ ions, $\mu_{H^{+}}$, we estimated as $k_{B}T\ln(c_{H^{+}}/c_{H^{+}}^{\mathrm{ref}});c_{H^{+}}^{\mathrm{ref}}=1\mathrm{mol}/L$, thus we have $\mu_{H^{+}}=-7k_{B}T\ln(10)$ (for more details see [59]).

### 4.3. Mean Field Theory

The Equation (3) expands as follows. The conformational entropy, *F*_conf_, accounts for the finite extensibility of the chain [67]:

(10) $$\begin{array}{c} \frac{F_{\mathrm{conf}}}{k_{B}T}=\frac{3}{2}\left[\frac{R^{2}/\left(Nb^{2}\right)-1}{{\left(1-R^{2}/\left(N^{2}b^{2}\right)\right)}^{d}}-\ln\left(\frac{R^{2}}{Nb^{2}}\right)\right], \end{array}$$

where the first term mimics Gaussian elasticity at small extensions and diverges when the chain is fully stretched. The logarithmic term accounts for the effect of chain compression. The parameter *d* > 0 characterizes the divergence behavior of the stretching energy; *b* is the chain Kuhn length (which we have chosen to be equal to *σ* from the CG model); and *R* is the end-to-end distance of a chain

(11) $$\begin{array}{c} R={\left(\frac{ANb^{3}}{\varphi }\right)}^{1/3}, \end{array}$$

where $A=3\sqrt{3}/4$ is the topological parameter of a diamond network, and *φ* is the polymer volume fraction. The molar density of the gel monomer units *ρ* is related to *φ* via coefficient: $\rho =\varphi /\left(N_{A}b^{3}\right)$.

The short-range non-electrostatic interactions, *F*_int_, are represented by the entropy of solvent (water) molecules and by the energy of polymer-solvent interactions defined by the Flory–Huggins parameter χ:

(12) $$\begin{array}{c} \frac{F_{\mathrm{int}}}{k_{B}T}=\frac{N}{\varphi }\left[\varphi_{w}\ln\varphi_{w}+\chi \varphi \varphi_{w}\right], \end{array}$$

where *φ*_w_ is the density of pure solvent without ions *φ*_w_ = 1 − *φ* − *φ*_+_ − *φ*_−_; *φ*_+_ and *φ*_−_ are the densities of mobile positive and negative ions [68], respectively.

The ionic part, *F*_ion_, is defined by the entropy and osmotic pressure of mobile ions as follows [22,34,69]:

(13) $$\begin{array}{c} \frac{F_{\mathrm{ion}}}{k_{B}T}=\frac{2c_{s}N}{\varphi }\left[1-\sqrt{1+{\left(\frac{\alpha \varphi }{2c_{s}}\right)}^{2}}\right]+N\ln\left(1-\alpha \right). \end{array}$$

*F*_int_ and *F*_ion_ depend on the gel ionization degree *α*, which in turn is calculated from the electroneutrality condition and Donnan equilibrium via

(14) $$\frac{\alpha }{1-\alpha }10^{-(\mathrm{pH}-pK)}=\sqrt{1+{\left(\frac{\alpha \varphi }{2c_{s}}\right)}^{2}}-\frac{\alpha \varphi }{2c_{s}}.$$

Thus, the free energy is a function of the gel volume fraction φ, so by taking the derivative of the hydrogel molar free energy with respect to the gel molar volume, $N_{A}b^{3}/\varphi =\rho$, we obtain the pressure applied to the gel

(15) $$p\left(\rho \right)=-\frac{N_{A}}{N}\frac{\partial F(\rho )}{\partial (1/\rho )}.$$

## Data Availability

Data is contained within the article or Supplementary Material.

## Supplementary Materials

The following supporting information can be downloaded at: https://www.mdpi.com/article/10.3390/gels9030259/s1. Figure S1: The pressure, p, as a function of the gel density, ρ, for different (a) pH–p*K*, (b,c) solvent quality ε and (d–e) salt concentration cs; Figure S2: The Maxwell constructions on pressure-extension curves of hydrogels with the same hydrophobicity ε = 0.5 and different pK and salt concentration. Approximate values of the transition pressures are written in the legends and represented by the dashed horizontal lines on the plots. Each value of transition pressure is printed on the state diagram in the main manuscript; Figure S3: Results of the bootstrap fit of pressure-extension curves of gels with pH–p*K* = 1.0 and the same hydrophobicity ε = 0.5 and at different salt concentrations. The result of the fitting is an approximate value of χ parameter written in the legend. Transition pressure, ptr is calculated via Maxwell construction (if possible). Thus the σ = 0.35, N = 50, b = 2, d = 1.36, χ = 0.96 are reasonable parameters to use in the analytical theory; Figure S4: Snapshots of hydrophobic gel with pH–p*K* = 1, ε = 0.5. Each row corresponds to a certain salt concentration cs and each column to certain gel density. Snapshots in colored frames show the gel in the coexistence region; Figure S5; Snapshots of hydrophobic gel with pH–p*K* = 1, ε = 0.7. Each row corresponds to a certain salt concentration cs and each column to certain gel density. Snapshots in colored frames show the gel in the coexistence region; Figure S6: Probability distribution function of local gel density for pH–p*K* = 1, ε = 0.5. Each row corresponds to a certain salt concentration cs and each column to a certain gel density; Figure S7: Probability distribution function of local gel density for pH–p*K* = 1, ε = 0.7. Each row corresponds to a certain salt concentration cs and each column to a certain gel density.
