# Supplementary material for: Modeling the Phase Transition in Hydrophobic Weak Polyelectrolyte Gels under Compression
Source: Gels. 2023 Mar 22;9(3):259. doi: 10.3390/gels9030259 (PMC10048452; doi:10.3390/gels9030259)
Supplement: Supplementary file 1 [file gels-09-00259-s001.zip › gels-2254340-supplementary.pdf]

gels

MDPI

Article

Modeling the Phase Transition in Hydrophobic Weak Polyelectrolyte Gels under Compression

Alexander D. Kazakov, Varvara M. Prokacheva, Oleg V. Rud, Lucie Nová and Filip Uhlík *

Department of Physical and Macromolecular Chemistry, Faculty of Science, Charles University, 12800 Prague, Czech Republic

* Correspondence: filip.uhlik@natur.cuni.cz.

Supporting information

1. Pressure-extension curves

Pressure-extension curves Changes in the salt concentration, pH−pK difference and/or deterioration of solvent quality leads to the phase separation in gels. The presence of such separation affects the hydrogel pressure-extension curve that appears to be non-monotonic dependence having a loop form. One of the possible ways to obtain an experimentally observable pressure-extension dependence is to employ the Maxwell construction. The final result of that procedure can be found in the main text of the article. Here, the original pressure-extension curves obtained from simulations are presented in Figure S1.

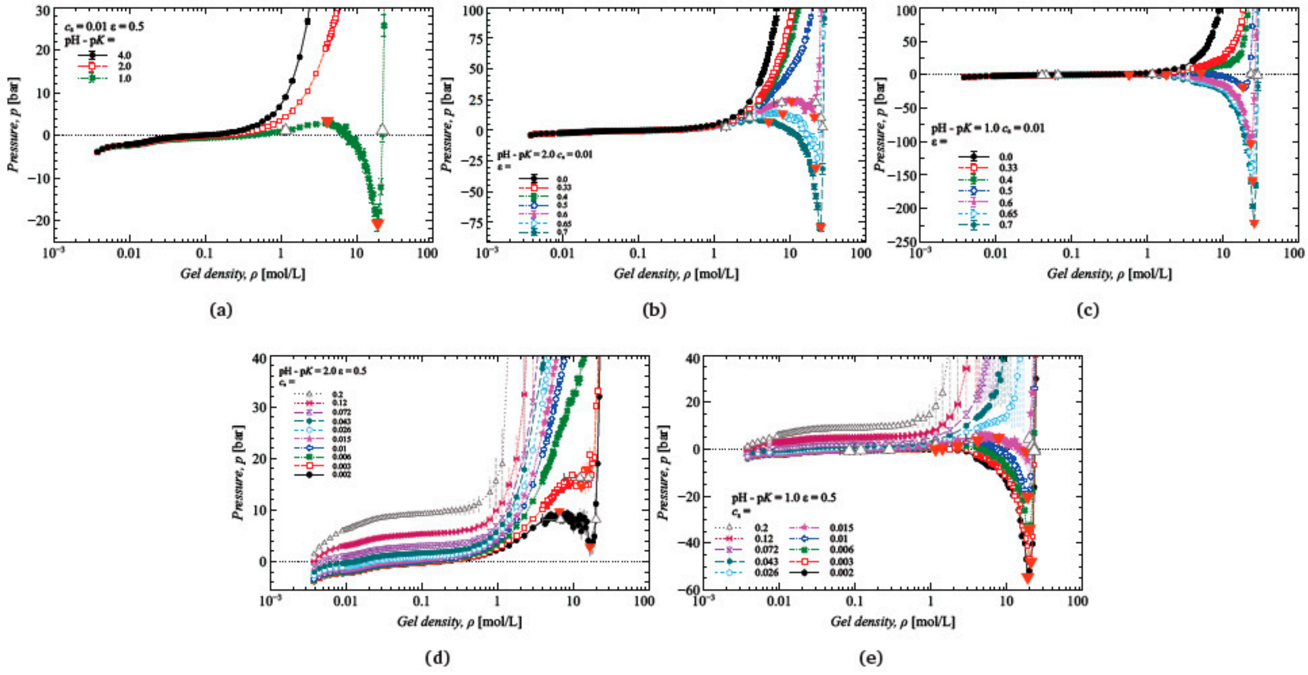

Figure S1. The pressure, p, as a function of the gel density, ϱ, for different (a) pH−pK, (b,c) solvent quality ε and (d–e) salt concentration c_s.

The less pH−pK is, the wider range of ε and/or c_s at which the phase separation takes place becomes. The points of the two phases coexistence are marked with white triangles, while the points of local maximum and minimum pressure—with orange triangles. Their coordinates were obtained during the Maxwell construction procedure explained in the following section. The connection of all white triangles gives the U-shaped coexistence

curve (binodal), while the connection of orange triangles gives a curve (spinodal) that separates the region of metastable states from the region of unstable states.

2. Maxwell construction procedure

The idea of the Maxwell construction is to find horizontal line drawn so that it cuts loops of equal areas above and below the line on a pressure-volume curve. The pressure corresponding to this line is a transition pressure, p_{tr}. The code (python script) is available on GitHub.

In Figure S2 the Maxwell construction over simulations data (purple symbols) are depicted for hydrophobic gel ε = 0.5 with pH−pK = 2 (Figure S2a,b) and pH−pK = 1 (Figure S2c–g) at different salt concentrations. As a result, the transition pressure and region of two phases coexistence were obtained. The left and right boundaries of coexistence region as well as local minimum and local maximum of the curve were marked with magenta, dark green, red, and green symbols, respectively. For cases of highly fluctuating simulation data, namely, the cases shown on the Figure S2b,g we have to use builtin interpolation method. As a conformation of reasonable interpolation we additionally provided original data from simulations (see Figure S2b,g). Thus we would like to show the robustness of simulation data by providing 10% uncertainty of pressure transition, which reflected in gel density ϕ. This uncertainty is depicted in white triangles of pressure-extension curves, ionization degree variation aswell as phase diagrams. Figure S2a,b and Figure S2c–g correspond to data in the phase diagram Figure S4a shown with red and black symbols, respectively.

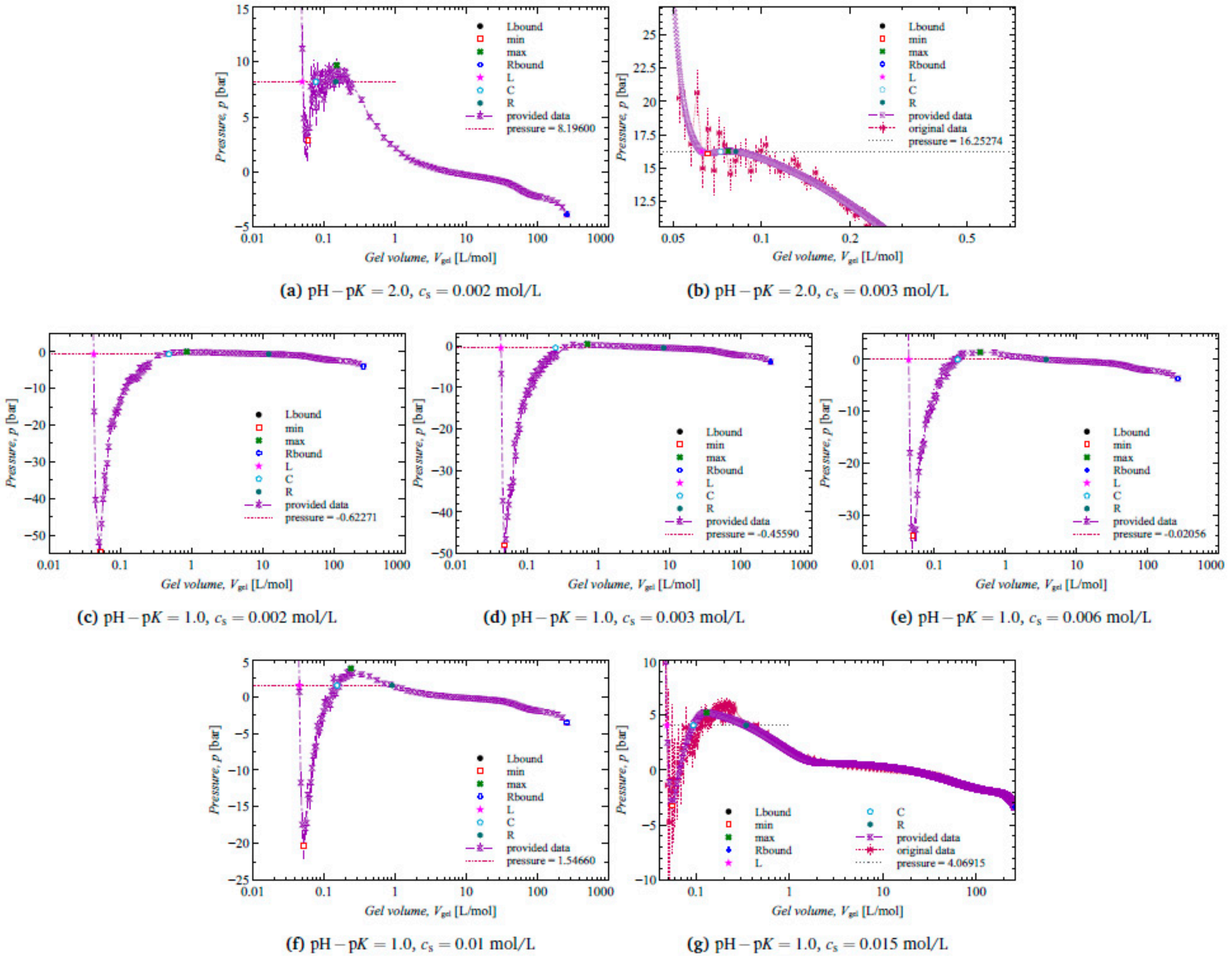

(a) pH − pK = 2.0, c_s = 0.002 mol/L **(b)** pH − pK = 2.0, c_s = 0.003 mol/L

(c) pH − pK = 1.0, c_s = 0.002 mol/L **(d)** pH − pK = 1.0, c_s = 0.003 mol/L **(e)** pH − pK = 1.0, c_s = 0.006 mol/L

(f) pH − pK = 1.0, c_s = 0.01 mol/L **(g)** pH − pK = 1.0, c_s = 0.015 mol/L

Figure S2. The Maxwell constructions on pressure-extension curves of hydrogels with the same hydrophobicity ε = 0.5 and different pK and salt concentration. Approximate values of the transition

pressures are written in the legends and represented by the dashed horizontal lines on the plots. Each value of transition pressure is printed on the state diagram in the main manuscript.

3. Fitting

In order to compare the results of MD simulations with results of the box-model we need the relation between χ and ε parameters that determine the solvent quality in each model. This we do by fitting simulation data by the analytical equation of the boxmodel Eq. 15 in main text.

In Figure S3 we show the fitting of MD data for the case of ε = 0.5 using analytical equation. The fits were performed using the least square method with bootstrapping, which we performed by means of 1000 random deletions of 95% of data points per curve. For each dataset realization we performed fit using Trust Region Reflective (TRF) method.

We allowed the value of χ to vary from 0.0 to 5.0, the value d to vary in range of 0.5 to 1.5, as well as the size of monomer σ in the range of 0.34 to 0.36 nm. The number of segments per chain N was kept fixed to the number of segments in CG simulation. During fitting *α*b was used instead of pH−pK (degree of ionization of a monomer in the bulk solution at given pH), which changes from 0 to 1. All the fittings resulted in the values of χ parameter ranging from 0.9 to 0.993, thus we conclude that the best choice of the parameter (fitting the CG data with ε = 0.5) is χ = 0.96.

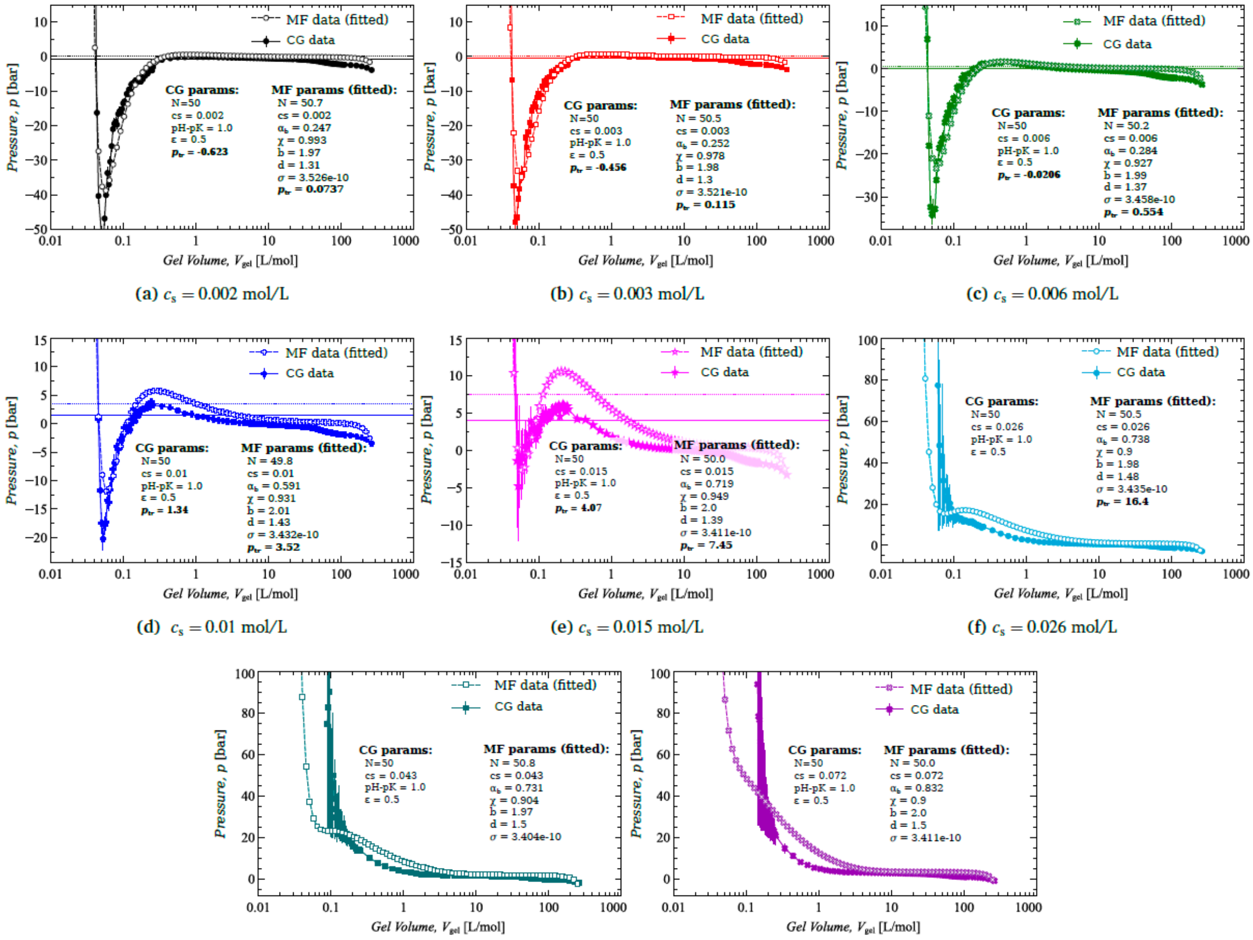

Figure S3. Results of the bootstrap fit of pressure-extension curves of gels with pH−pK = 1.0 and the same hydrophobicity ε = 0.5 and at different salt concentrations. The result of the fitting is an approximate value of χ parameter written in the legend. Transition pressure, p_{tr} is calculated via Maxwell construction (if possible). Thus the σ = 0.35, N = 50, b = 2, d = 1.36, χ = 0.96 are reasonable parameters to use in the analytical theory.

4. Snapshots

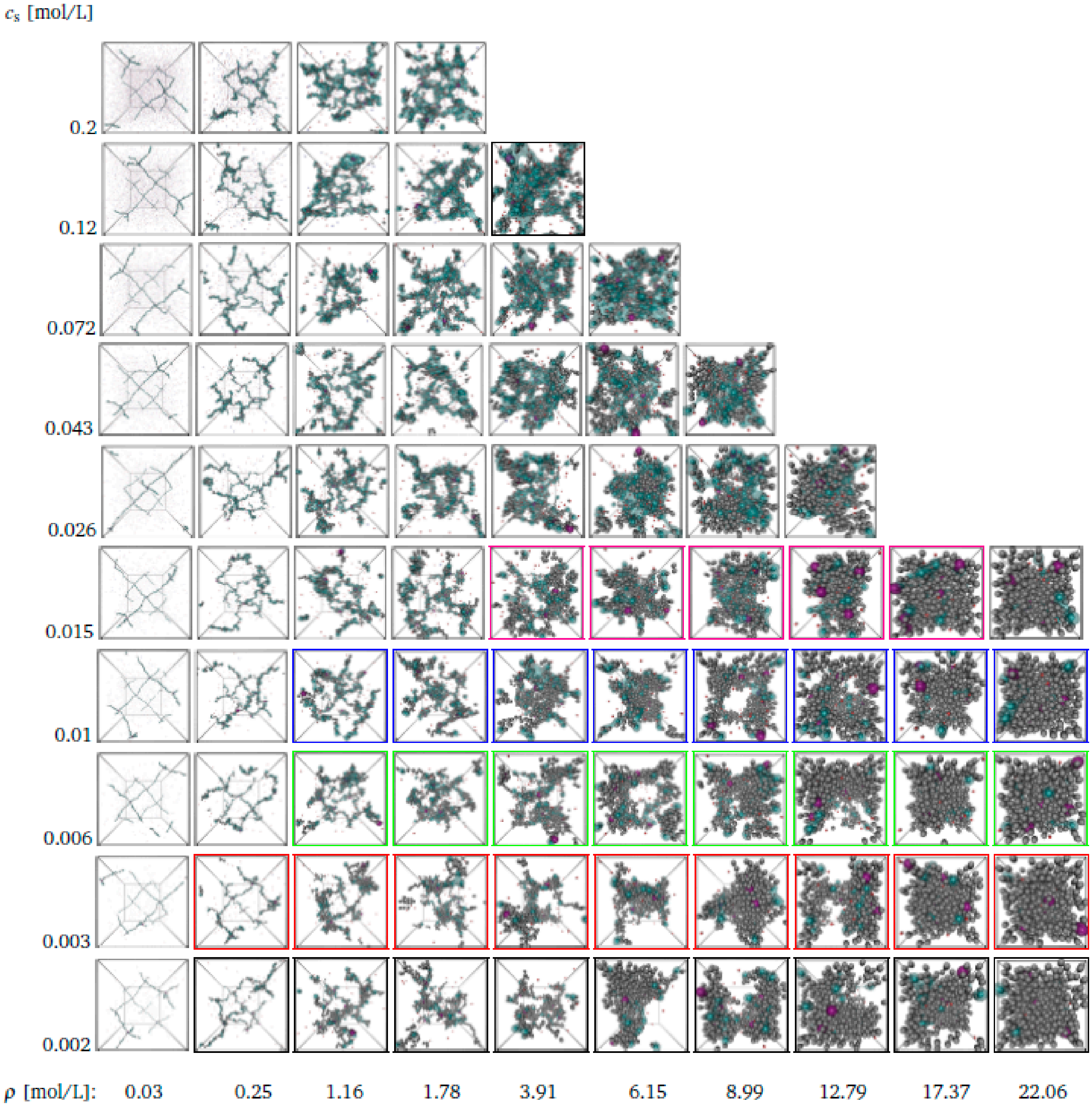

Figure S4. Snapshots of hydrophobic gel with pH−pK = 1, ε = 0.5. Each row corresponds to a certain salt concentration c_s and each column to certain gel density. Snapshots in colored frames show the gel in the coexistence region.

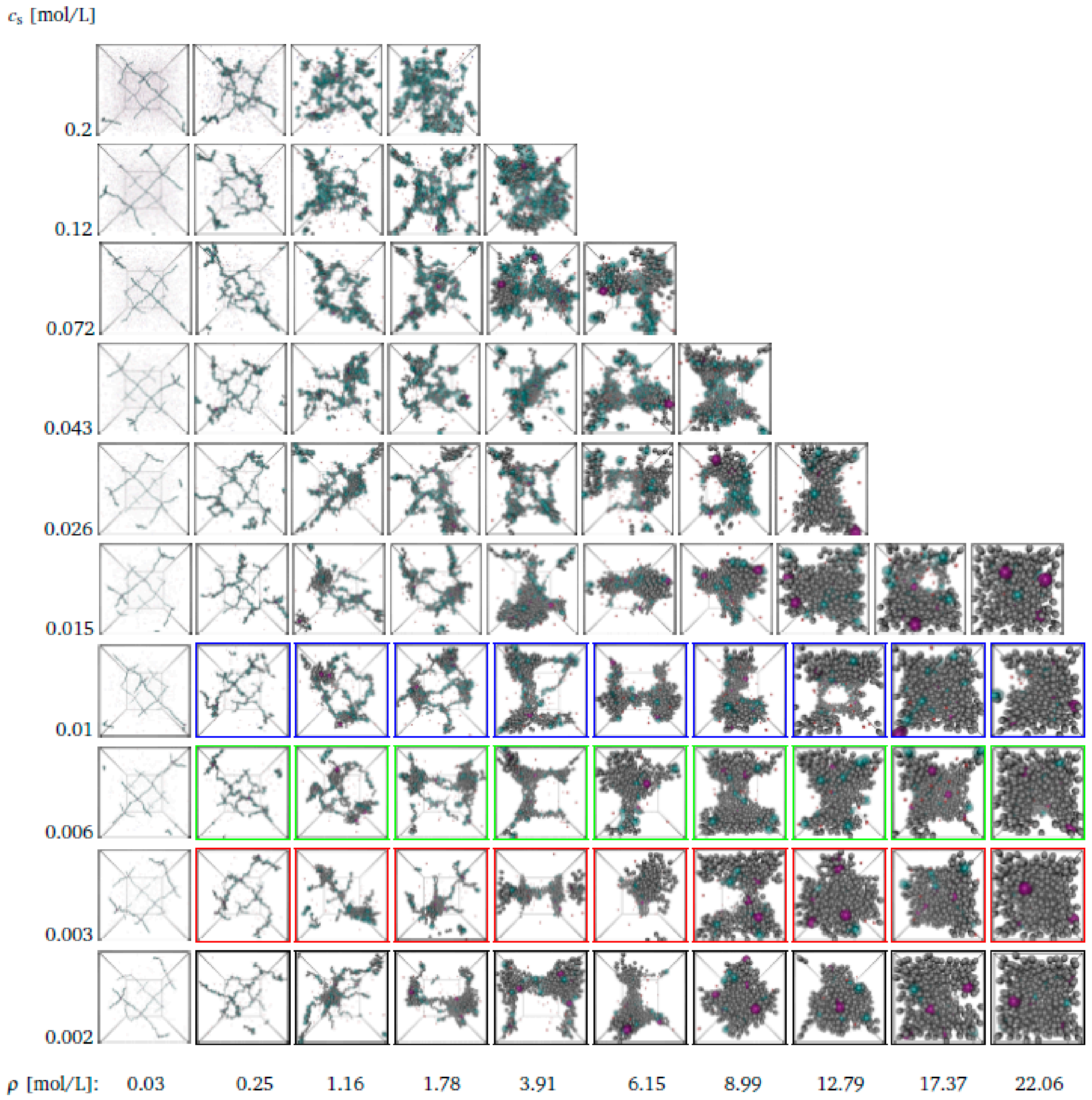

Figure S5. Snapshots of hydrophobic gel with pH−pK = 1, ε = 0.7. Each row corresponds to a certain salt concentration c_s and each column to certain gel density. Snapshots in colored frames show the gel in the coexistence region.

5. Local density distributions

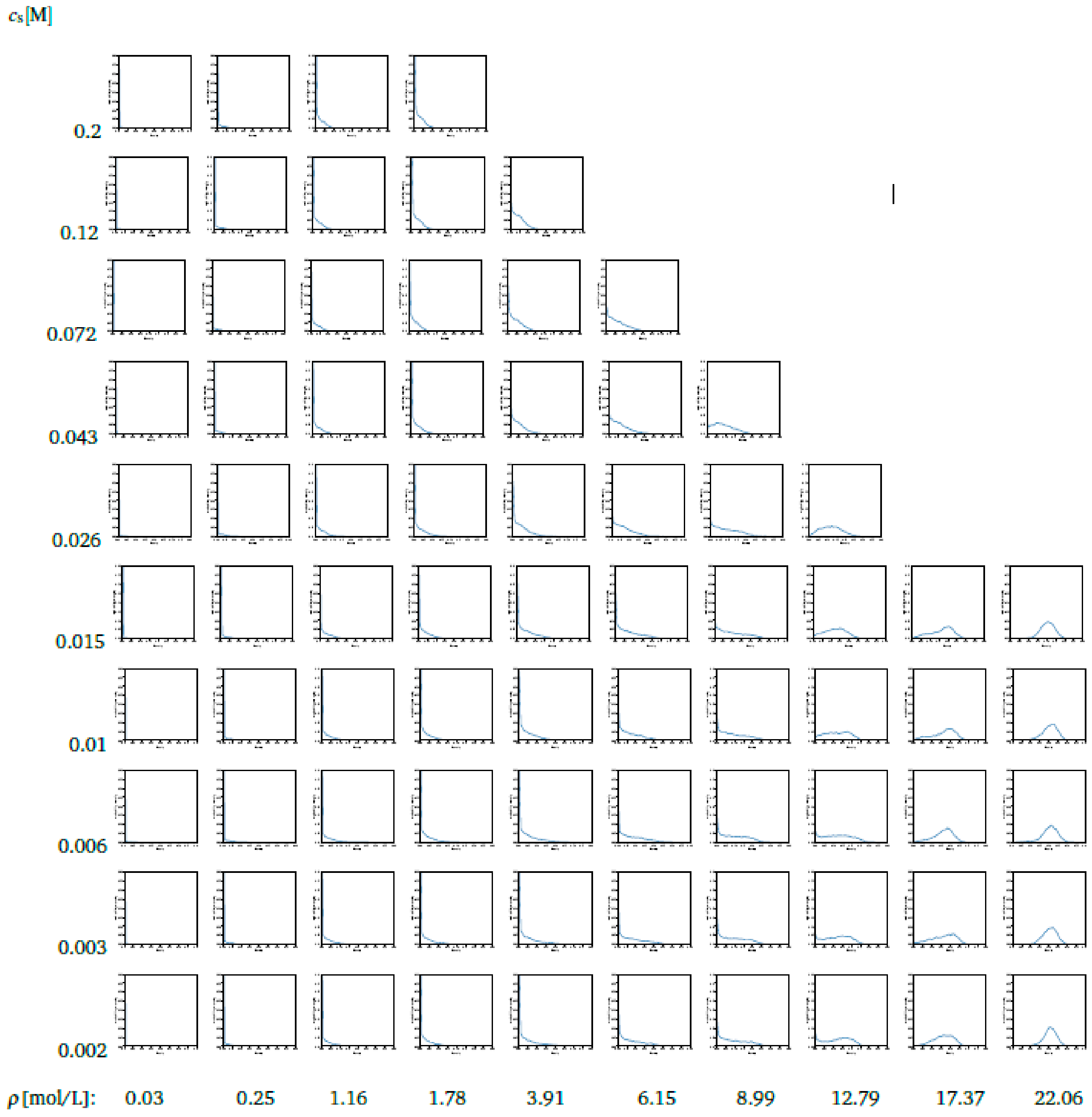

Figure S6. Probability distribution function of local gel density for pH−pK = 1, ε = 0.5. Each row corresponds to a certain salt concentration c_s and each column to a certain gel density.

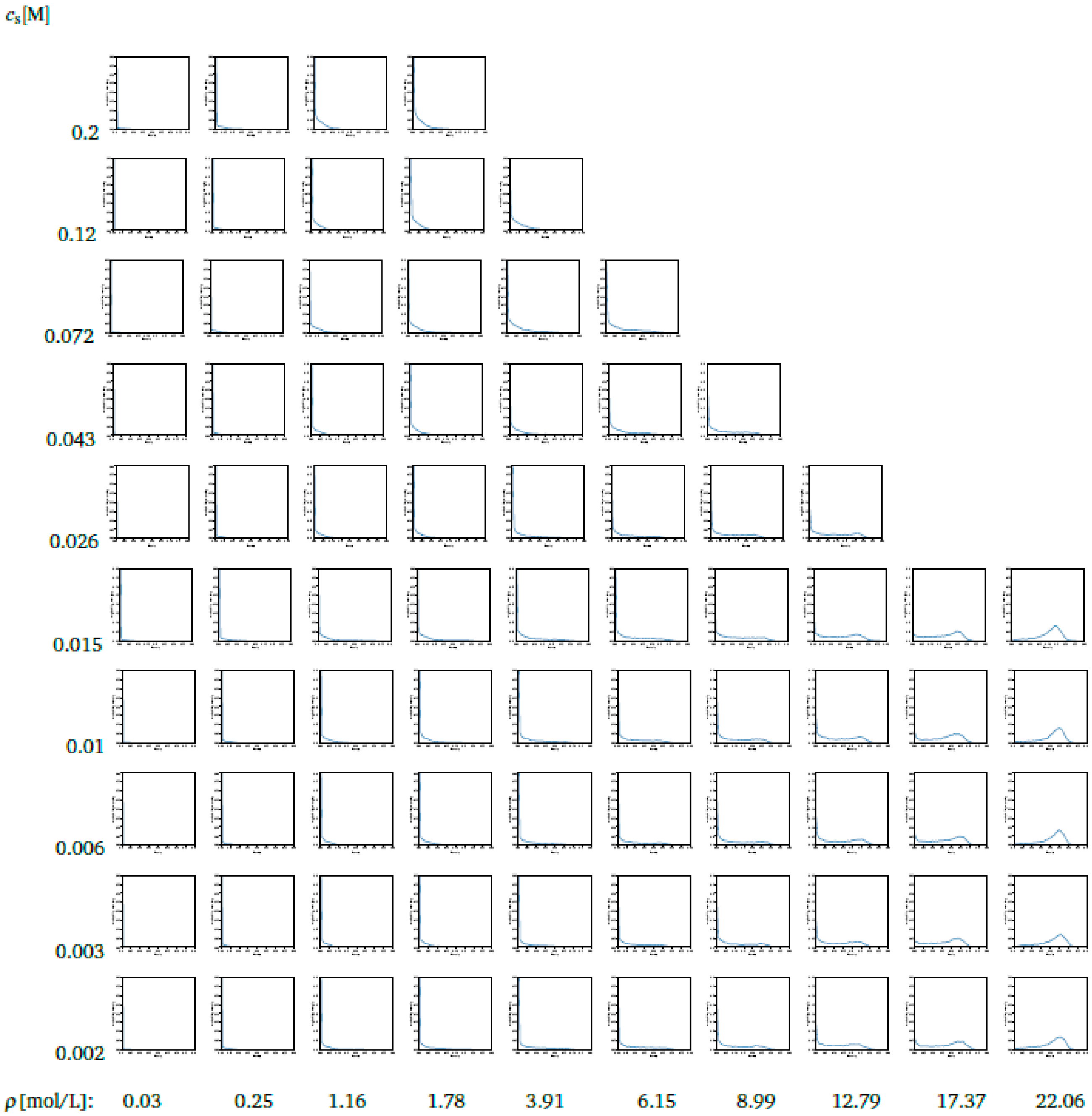

Figure S7. Probability distribution function of local gel density for pH−pK = 1, ε = 0.7. Each row corresponds to a certain salt concentration c_s and each column to a certain gel density.
